# Event Knowledge Modulates Real‐Time Mental Representations of Object State‐Change

**DOI:** 10.1111/cogs.70165

**Published:** 2026-01-20

**Authors:** Sarah Hye‐yeon Lee, Elsi Kaiser

**Affiliations:** ^1^ Department of Linguistics University of Pennsylvania; ^2^ Department of Linguistics University of Southern California

**Keywords:** Change‐of‐state, Event knowledge, Event representation, Grammatical aspect, Language comprehension, Object state

## Abstract

The present study examines how real‐world event knowledge and grammatical aspect guide event comprehension. Specifically, we tested whether real‐world knowledge about the likelihood of state‐change (e.g., wine glasses usually crack when dropped but plastic cups do not) modulates the object state representations that people construct while reading perfective and imperfective sentences. Participants read “rebus” sentences in perfective and imperfective aspect, presented one word at a time, self‐paced. In each sentence, the object was replaced by an image of the object that is either likely or unlikely to undergo state‐change (e.g., Carlos was dropping/dropped a *wine glass*/*plastic cup* …), depicted in their initial (intact) or end (changed) states. Reaction times to images indicate that real‐world knowledge about the likelihood of state‐change is recruited when comprehenders construct mental models of events described as completed (*perfective* aspect, e.g., *dropped*) as well as events described as ongoing (*imperfective* aspect, e.g., *was dropping*). Results also indicate that perfective aspect increases the accessibility of both the initial and end states of objects, compared to imperfective aspect. Overall, these results demonstrate that both non‐linguistic information grounded in real‐world event knowledge as well as linguistic cues about the temporal structure of events guide how comprehenders dynamically update mental representations of object states in real‐time.

## 1 Introduction

Event understanding is not an isolated process; it is deeply tied to our real‐world knowledge, including prior knowledge about how events typically take place in the world. During language comprehension, comprehenders integrate information from the linguistic signal with prior world knowledge to construct a mental understanding of the situation (e.g., Altmann & Mirković, 2009; Elman, 2009; Sanford & Garrod, 1998; van Dijk & Kintsch, 1983), and this mental representation in turn rapidly affects comprehension (e.g., McRae, Spivey‐Knowlton, & Tanenhaus, 1998; Metusalem et al., 2012). There is robust psycholinguistic evidence that comprehenders rely on real‐world event knowledge to dynamically update their expectations about the upcoming linguistic material (e.g., Altmann & Kamide, 1999; Bicknell, Elman, Hare, McRae, & Kutas, 2010; Ferretti, McRae, & Hatherell, 2001; Hare, Jones, Thomson, Kelly, & McRae, 2009; Matsuki et al., 2011; McRae & Matsuki, 2009; McRae, Hare, Elman, & Ferretti, 2005).

Event knowledge encompasses typical actors, locations, and the manner in which situations unfold. This includes, for example, knowledge about typical thematic roles associated with an action (e.g., *a detective* is a likely agent of a spying action), which can influence anticipatory eye movements (Knoeferle & Crocker, 2006). However, event knowledge encompasses more than just information about typical participants and locations; it also includes knowledge about the *changes* that event participants undergo (Altmann & Ekves, 2019). For instance, we learn throughout our lives that wine glasses are likely to break when dropped, whereas plastic cups are not, and that eggs typically crack when thrown, unlike rocks. What remains less clear, and is the focus of this study, is how and to what extent this latter, less‐studied aspect of real‐world event knowledge—that is, the changes that event participants typically undergo—is accessed during real‐time language comprehension.

Of relevance to this question is a recent theory of mental event representations, the Intersecting Object Histories account (Altmann & Ekves, 2019), which emphasizes the importance of objects and their states in event comprehension. This theory suggests that understanding an event involves tracking objects and their states, a claim supported by a growing body of research (Hindy, Altmann, Kalenik, & Thompson‐Schill, 2012; Horchak & Garrido, 2021; Kang, Eerland, Joergensen, Zwaan, & Altmann, 2020; Lee & Kaiser, 2021; Misersky, Slivac, Hagoort, & Flecken, 2021; Prystauka, Wing, & Altmann, 2024; Solomon, Hindy, Altmann, & Thompson‐Schill, 2015). Recent studies have examined several factors that can modulate the representation of object states during comprehension, such as grammatical tense and the degree of state‐change implied by the verb (e.g., “The woman *chose/stepped on* a banana”; Kang et al., 2020), the impact caused by items differing in weight (e.g., “You drop a *balloon/bowling ball* on a tomato”; Horchak & Garrido, 2021), lexical and discourse‐level information (Lee & Kaiser, 2021), and grammatical aspect (Misersky et al., 2021). For example, comprehending the sentence “The woman stepped on a banana” requires activating both the initial (intact) state and the end (changed) state of a banana—the consequence of it being squashed by the woman.

Prior work has examined object state‐change using clearly distinct situations—for instance, events involving objects like *bananas* or *eggs* that are unambiguously going to be altered (e.g., in a stepping‐on event) versus *coins* that are not (Hindy et al., 2012). Other work has also compared state‐change verbs that semantically entail a change (e.g., *inflate* a balloon, *chop* an onion) against minimal‐change verbs that neither entail nor imply any change (e.g., *notice* a balloon, *weigh* an onion; Hindy et al., 2012; Kang et al., 2020; Misersky et al., 2021; Prystauka et al., 2024; Solomon et al., 2015). However, what remains less clear is how real‐time comprehension of event descriptions is affected when the likelihood of state‐change is less categorical, varying as a function of the object's properties. For example, stepping on a banana will surely squash it and stepping on a coin will do no harm, but what if the woman stepped on a pinecone? Is the pinecone intact or crushed? We explore this question using verbs whose lexical semantics imply, but do not semantically entail, an intrinsic change‐of‐state (e.g., *to hammer*, *to drop*). These verbs elicit weaker state‐change representations than verbs like *break* or *shatter* that semantically entail such changes (Lee & Kaiser, 2021). Crucially, using verbs that do not entail change allows us to investigate whether and how *object properties* modulate the mental representation of state‐change. Investigating potential effects of object properties would not be possible with verbs that entail change, as they trigger state‐change interpretations regardless of the object involved.

Furthermore, it is unclear how real‐world event knowledge influences the processing of event descriptions in real‐time. Influential prior work (e.g., Kang et al., 2020; Madden & Zwaan, 2003; Prystauka et al., 2024) has probed comprehenders’ post‐sentential event representations, by asking them to explicitly judge whether the object displayed was mentioned in the sentence that they read. This method allows the researcher to assess whether the depicted object state is congruent with the event representation built based on the full sentence. However, it does not provide direct information about how the object state representations that comprehenders construct are updated in real‐time during online sentence comprehension. Contributing to this question is one of the aims of our work.

In addition to real‐world event knowledge, object state‐change is linked to the temporal structure of events, for example, whether an event is ongoing or completed. This is because object state‐change is temporal in nature—that is, the state of an object can change over the course of an event (e.g., from the beginning of an event to the end). Many languages use imperfective aspect (e.g., *Carlos was dropping the cup*) and perfective aspect (e.g., *Carlos dropped the cup*) to express this kind of temporal‐semantic information about events.

While imperfective aspect emphasizes the ongoing nature of an event, perfective aspect focuses on the holistic nature of an event (e.g., Comrie, 1976; Dowty, 1986). Imperfective aspect is compatible with an interpretation where the event was interrupted midway, whereas perfective aspect signals that the entire event took place, from beginning to end. Prior experimental work showed that aspectual cues modulate the construction of object state representations during event comprehension, with perfective aspect emphasizing event boundaries and imperfective shifting focus away from event boundaries (Madden‐Lombardi, Dominey, & Ventre‐Dominey, 2017; Magliano & Schleich, 2000; Misersky et al., 2021). For example, in Misersky et al.’s (2021) ERP study, participants read English sentences where the action denoted by the verb phrase inevitably leads to (i.e., entails) a change‐of‐state (e.g., *chop an onion*), in perfective (*chopped*) or imperfective aspect (*was chopping*). Participants then saw a picture that showed either an object that had undergone substantial state‐change (a chopped onion) or no state‐change (an onion in its original unchopped state) and indicated whether the pictured object was mentioned in the sentence. Among their findings, Misersky et al. observed that state‐change pictures elicited a higher‐amplitude P300 after the perfective (*chopped*) than after the imperfective (*was chopping*) sentences. Given that the P300 can be understood to reflect attention and stimulus evaluation (Polich, 2007), Misersky et al. interpret this as evidence that perfective aspect on the verb leads to specific attentional focus on object state‐change and event completion. While focus on the initial state is also in principle compatible with the holistic event representation cued by perfective aspect, Misersky et al. did not find empirical evidence for perfective aspect focusing initial states. Overall, these results show that grammatical aspect modulates attention, with perfective aspect focusing attention on event boundaries, which include, at least, the object's end state in state‐change events. While these findings revealed a close link between perfective aspect and object state‐change, they leave open the question of whether such an effect extends to “ambiguous” verbs such as *to drop* and *to hammer* whose lexical semantics allow for, but do not entail, state‐change.

### 1.1 The Current Study

In this work, we are interested in whether and how fine‐grained *real‐world event knowledge about the likelihood of state‐change* (e.g., wine glasses are likely to get damaged when dropped, but plastic cups are less likely to) and *grammatical aspect* (imperfective vs. perfective) guide the mental representations comprehenders build in real‐time based on linguistic input. In order to tap into comprehenders’ real‐time mental representations of object states in as direct a way as possible, we used images that depicted whether an object has undergone a state‐change or not and presented them mid‐sentence.

Our first aim is to investigate how fine‐grained real‐world event knowledge about the likelihood of state‐change influences the object state representations that comprehenders construct during real‐time sentence comprehension. We know that wine glasses are more likely to break when dropped than are plastic cups. Does this knowledge about the likelihood of state‐change influence the mental representations of object states that we construct (e.g., a damaged wine glass vs. an intact plastic cup) when processing linguistic input describing an event that potentially involves a state‐change (e.g., a dropping event involving a wine glass vs. a plastic cup)?

Second, we investigate how grammatical aspect in the verbal domain modulates the object state representations during incremental language comprehension. We are interested in whether initial and end states of an object are more accessible in perfective versus imperfective aspect. If perfective aspect elicits a holistic event representation with focus on event boundaries, both initial and end states should be more accessible in perfective aspect, compared to imperfective aspect. In addition, we ask whether comprehenders activate real‐world knowledge about the likelihood of state‐change in different ways when the verb presents an event as completed (perfective aspect), compared to when the verb presents the event as ongoing (imperfective aspect). We test English, a language where the verb typically precedes the object, thus allowing us to test how aspectual information encoded in the verbal domain shapes real‐time processing of visually depicted object states.

## 2 Experiment

To investigate how real‐world knowledge and grammatical aspect affect comprehenders’ mental representations of object states, we used an image‐based rebus paradigm, similar to Madden and Therriault (2009; see also Madden‐Lombardi et al., 2017). In the rebus paradigm, participants read sentences word‐by‐word, but the critical word (or words) is replaced with an image of it (e.g., … open a *image of a wine bottle*). (The term “rebus” refers to the fact that the stimuli are a combination of text and images.) Using images allowed us to probe visual representations of object states. In Madden and Therriault (2009; see also Madden‐Lombardi et al., 2017, for a related study in French), the object in each transitive subject–verb–object English sentence was replaced by an image. Crucially, the images were either congruent or incongruent with the aspectual information signaled by the preceding verb. For example, for a perfective sentence, an image of an already‐opened wine bottle is aspect‐congruent, but an image of a not‐yet‐opened bottle is incongruent. This “rebus” paradigm—unlike the post‐sentential picture verification task used in prior research (e.g., Hindy et al., 2012; Kang et al., 2020; Madden & Zwaan, 2003)—allows researchers to tap into how comprehenders integrate visual depictions of object states in real‐time.

In particular, for languages with subject–verb–object order, one can present different kinds of object images after verbs and test for effects of grammatical aspect. By measuring how quickly participants process the object images, this method taps into comprehenders’ object state representations during incremental sentence processing. The basic idea is that images congruent with the mental model that the comprehender has constructed thus far in the sentence should be easier to integrate and yield faster reaction times (RTs) than images incongruent with the mental event representation. In other words, RTs provide an indication of the cognitive processes taking place as visual stimuli are integrated into comprehenders’ event representations during real‐time sentence processing; by manipulating the visual stimuli, we can detect what kinds of event representations participants are constructing.

### 2.1 Method

#### 2.1.1 Participants

Adult native speakers of English were recruited on the internet via Prolific. Participants were compensated $4. A total of 242 native English speakers participated, and all were included in the final analysis. This is because no one scored below the 80% accuracy threshold on the comprehension questions (mean accuracy = 97.70%). (All exclusion criteria were determined before data analysis was conducted.) The experiment was reviewed and approved by the University of Southern California Institutional Review Board.

#### 2.1.2 Design and materials

We manipulated (a) object type (likely vs. unlikely to undergo state‐change), (b) the visually depicted object state (no‐state‐change vs. state‐change), and (c) grammatical aspect (perfective vs. imperfective). The object type manipulation concerned whether the object was likely or unlikely to undergo state‐change as a result of the described action (e.g., dropping a wine glass vs. a plastic cup). The objects for each condition were selected from a pool of objects identified by several native English‐speaking consultants as likely or unlikely to undergo state‐change as a result of the action described by the verb. Crucially, the object word(s) (e.g., wine glass, plastic cup) were replaced by an image of that object. As regards object state, we tested images depicting *no‐state‐change* (e.g., intact glass/cup) and images depicting *state‐change* (e.g., damaged glass/cup). The *no‐state‐change* images correspond to initial states, and *state‐change* images correspond to end states. Finally, we manipulated the grammatical aspect; sentences were presented in the perfective or imperfective aspect. The English simple past was used to represent perfective aspect (e.g., *dropped*), and the (past) progressive was used to represent imperfective aspect (e.g., *was dropping*, see Table 1). (All sentences were in past tense.)

**Table 1:** Sample target item for the experiment

	Object State	Pre‐Image *Text*	Object Type		Post‐Image *Text*
Grammatical Aspect			Unlikely to Undergo State Change	Likely to Undergo State Change	
Imperfective	No‐state‐change	*Carlos was dropping the*	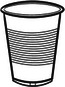	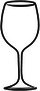	*at the busy bar*
	State‐change	*Carlos was dropping the*	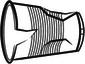	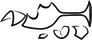	
Perfective	No‐state‐change	*Carlos dropped the*	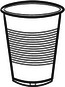	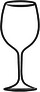	
	State‐change	*Carlos dropped the*	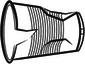	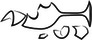	

In the images, the same object was depicted either in its intact (no‐state‐change) or changed state. That is, the only difference between the no‐state‐change and state‐change image types was their states, for example, an intact wine glass and a damaged wine glass. The images were drawn specifically for this study, to ensure that objects and object states were clearly recognizable. See Table 1 for a sample target item. The study included 24 target items in total. In addition to the 24 target items, the study included 32 filler items. Filler items also had one of the words replaced by an image. Our materials are available at this link: https://osf.io/rgx2a/.

As this study had a 2 × 2 × 2 design, eight lists were created and presented to participants using a standard Latin square design: Each participant saw each of the 24 targets only once, and each of the eight conditions appeared the same number of times on any given list. Each of the eight lists contained the same set of filler items, pseudo‐randomly distributed throughout the list.

#### 2.1.3 Procedure

The experiment was hosted online on PennController IBEX (Zehr & Schwarz, 2018; https://www.pcibex.net/). Participants read the sentences word‐by‐word in a moving‐window self‐paced reading paradigm. Crucially, images were displayed as if they were words, that is, in their appropriate linear location in the sentence. Participants were instructed ahead of time that they would see both words and images and that they should treat these images as part of the sentences in which they occur (see also prior work using the rebus paradigm, e.g., Madden & Therriault, 2009). With each keypress, the currently displayed word/image disappeared, and the next word/image was displayed. RTs to each word/image were recorded.

Before the main experiment, participants completed three practice trials, to help them become familiar with the rebus paradigm. Each trial was followed by a comprehension question with two answer choices. The questions asked about different sentence regions, namely the location, the agent, or the adjectival description in the post‐image text. To avoid triggering strategic processing, none of the questions asked about the object's state. Incorrect responses to comprehension questions triggered an error feedback message.

### 2.2 Predictions

#### 2.2.1 Event knowledge violation penalty

We investigate whether real‐world event knowledge about the likelihood of state‐change mediates the construction of object state representations during incremental processing. If it does, encountering an image of an object state‐change that is relatively unlikely (e.g., seeing a damaged plastic cup after encountering the verb *drop*) should result in an overall higher processing load than a more likely object state‐change (e.g., a broken wine glass) due to difficulty integrating the visually presented information (the image of the broken plastic cup) into the comprehender's current mental event model. We call this the *event knowledge violation penalty*. The difficulty of recognizing an unlikely changed state is related to its atypical features, and our real‐world event knowledge about the likelihood of state‐change is related to our direct sensory experience (e.g., how often we see different kinds of objects in their changed vs. unchanged states). Crucially, these kinds of atypicality effects are known to be modulated by surrounding linguistic context: when the atypical features are part of the event structure associated with the sentence, those features can be more activated (Prystauka et al., 2024). For instance, a deflated balloon is an atypical state of a balloon, but Prystauka et al. showed that the deflated state was more accessible after state‐change verbs (“inflate”) than after minimal‐change verbs (“choose”), because a deflated balloon is part of the representation of the inflating event. In other words, linguistic context modulates the accessibility of atypical states: It can be boosted when the verb entails a state‐change.

Building on these insights, our work explores what happens in *ambiguous* contexts where the verb in the linguistic input allows for, but does not entail, state‐change. Specifically, does real‐world knowledge about the likelihood of state‐change guide processing in such contexts? Does an event knowledge violation penalty—that is, processing difficulty with unlikely state changes—occur in a linguistic context where the event structure elicited by the verb does not entail a state‐change?

If the answer is “yes” and there is an event knowledge violation penalty in these contexts, RTs to state‐change images should be affected by object type, with unlikely state‐change images (e.g., damaged plastic cup) eliciting longer RTs across the board than likely state‐change images (e.g., damaged wine glass). In contrast, no‐state‐change images (e.g., intact plastic cup, intact wine glass) should not be systematically affected by object type. Thus, we expect an interaction between object type (unlikely vs. likely to undergo state change) and object state (no‐state‐change vs. state‐change): There should be an effect of object type in state‐change images, but not in no‐state‐change images.

However, there is a possibility that real‐world event knowledge (and the atypicality associated with it) has no effect on the construction of object state representations during incremental processing (no event violation penalty) when the changed object states are part of the event structure elicited by the linguistic input (see Prystauka et al., 2024, for related findings). In other words, perhaps the event structure evoked by the linguistic input is strong enough to activate even those state‐changes that are less likely. If this is the case, then whether an object is (un)likely to undergo state‐change should not affect RTs. Any RT differences between state‐change images of objects likely versus unlikely to undergo state‐change should be comparable to any RT differences between no‐state‐change images of objects likely versus unlikely to undergo state‐change (i.e., no interaction between object type and object state).

(In order to assess whether real‐world knowledge about the likelihood of state‐change has an effect, it was necessary for us to use different objects, e.g., a cup and a glass. Due to the objects being intrinsically different, we may find differences between the two types of no‐state‐change images that are independent of the likelihood to undergo a change‐of‐state. However, these differences are not crucial for our predictions.)

#### 2.2.2 Aspect and object state‐change

We also examine how different object states are integrated into the event model constructed from different temporal viewpoints, as signaled by perfective versus imperfective aspect. Given prior findings that perfective aspect, unlike imperfective aspect, focuses attention on the boundaries of events (e.g., Madden‐Lombardi et al., 2017; Magliano & Schleich, 2000; Misersky et al., 2021), it is possible that both initial and end states are integrated faster into the event model when the sentence is in perfective aspect, compared to imperfective aspect. However, it is also possible that there is no such aspect difference when the verb does not semantically entail state‐change, as is the case with our verbs.

In addition to testing for an overall difference between perfective versus imperfective aspect, we also ask whether the availability of event knowledge about the likelihood of state‐change (the event knowledge violation penalty) is modulated by grammatical aspect. One possibility is that event knowledge about state‐change is more readily available in perfective than in imperfective aspect, given claims that perfective aspect, unlike imperfective aspect, focuses attention on state‐change (e.g., Madden‐Lombardi et al., 2017; Misersky et al., 2021). If this is the case, the event knowledge violation penalty associated with the difficulty of integrating an unlikely object state‐change should be amplified in perfective aspect, compared to imperfective aspect, leading to an interaction between grammatical aspect and object type, specifically within the state‐change image conditions. However, it is also possible that the event knowledge violation penalty persists regardless of linguistic cues. If this is the case, the RT difference between likely and unlikely state‐change images in perfective aspect sentences should not be any greater than the RT difference between likely and unlikely state‐change images in imperfective aspect sentences (i.e., no interaction between object type and object state within state‐change images).

### 2.3 Data processing and analysis

When analyzing RTs to the images, we first removed RTs under 100 ms and over 5000 ms. This removed 19 datapoints (0.03% of the data). No datapoint was more than 3 SDs away from a participant's mean RT to the object image, so no data were excluded due to this criterion. This left us with 5789 datapoints for the final analysis. (The criteria for outlier removal were determined prior to data analysis.) After outlier removal, RTs were log‐transformed to reduce skewness and normalize the distribution.

For statistical analyses, we used linear mixed effects models with RT as the dependent variable, and Grammatical aspect (contrast‐coded; imperfective = 0.5, perfective = −0.5), Object type (contrast‐coded; likely to undergo state change = 0.5, unlikely to undergo state change = −0.5), Object state (contrast‐coded; state‐change = 0.5, no‐state‐change = −0.5), Grammatical aspect × Object type interaction, Grammatical aspect × Object state interaction, and Object type × Object state interaction as fixed effects. As random effects, we entered intercepts for participants and items, as well as by‐participant and by‐item random slopes. They were then reduced (starting with by‐item effects) via model comparison, wherein only random effects that contributed significantly to the model (*p* < .05) were included (Baayen, Davidson, & Bates, 2008).

Models were estimated using the *lme4* package (version 1.1.26; Bates, Mächler, Bolker, & Walker, 2015) and *lmerTest* (version 3.1.3; Kuznetsova, Brockhoff, & Christensen, 2017) in the R software environment (R Development Core Team, 2019). For planned comparisons comparing no‐state‐change conditions and state‐change conditions, we fit models that had Grammatical aspect, Object type, and their interaction as fixed effects. For planned comparisons comparing the perfective aspect conditions and the imperfective aspect conditions within state‐change images, we fit models with Object type as the fixed effect. For these analyses, we again used the maximal random effect structure justified by model comparison, and we used the same R packages. Our data and analysis scripts are available on our OSF page at: https://osf.io/rgx2a/.

Our predictions and analyses crucially depend on comparing RTs to different images (e.g., a damaged plastic cup vs. a damaged wine glass). This approach aligns with earlier studies (e.g., Kang et al., 2020; Madden & Therriault, 2009; Madden‐Lombardi et al., 2017) that also compared RTs to different images within the same linguistic context. It is a crucial part of our prediction that RTs to images depicting changed states of different types of objects (e.g., likely vs. unlikely to undergo state‐change) would be different—if the event knowledge violation penalty holds.

### 2.4 Results

**Fig. 1:**
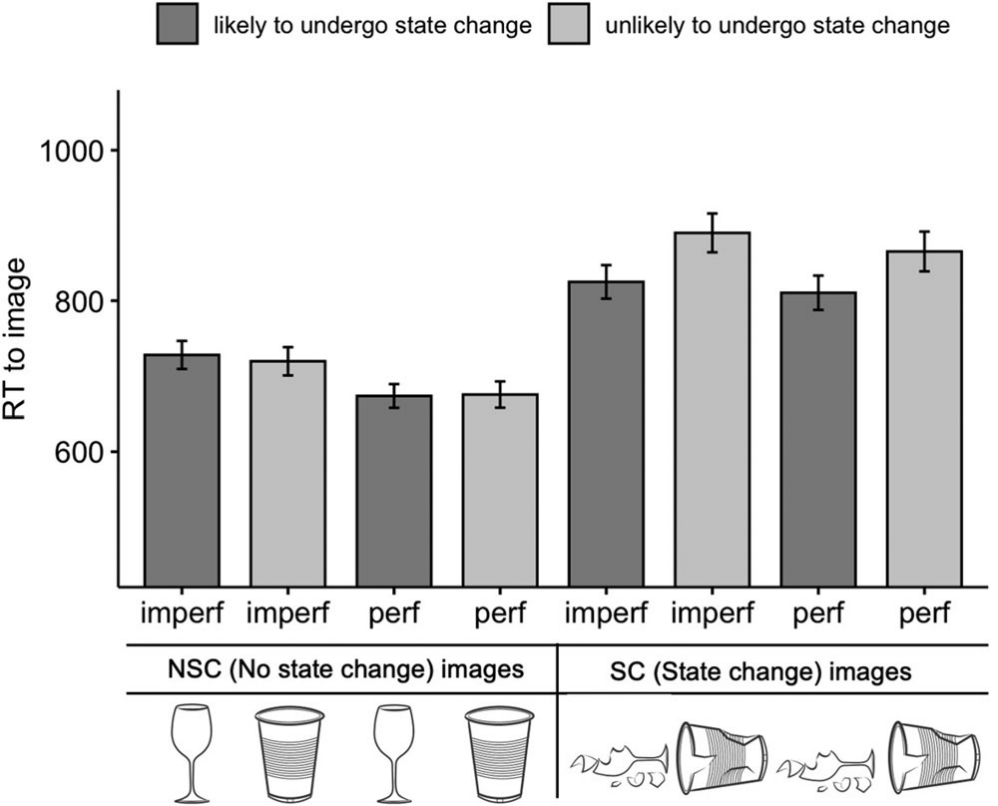
Average raw reaction times (RTs) to the image by condition (ms) (error bars represent ± 1 *SE*).

Fig. 1 shows the average RTs at the image, by condition. (All statistical analyses were conducted with log‐transformed RTs. However, for ease of interpretation, raw RTs are presented in Fig. 1. The patterns observed in the raw RTs were consistent with those found using log‐transformed RTs.) Statistical analyses are reported in Table 2. Overall, as can be seen in Fig. 1, RTs to the state‐change images were longer than RTs to the no‐state‐change images (*p* < .001). This main effect of object state is expected as the state‐change images are divergent from prototypical depictions of the object, as mentioned above, but this is not part of our theoretical predictions. Therefore, we do not discuss it further.

**Table 2:** Linear mixed model results

	*β*	*SE*	df	*t* value	*Pr* (>|*t*|)
(Intercept)	6.41	0.05	96.13	134.09	<0.001^***^
Object type	−0.02	0.01	5516.98	−1.69	0.09
Grammatical aspect	0.04	0.01	5517.05	3.99	<0.001 ^***^
Object state	0.13	0.01	5516.99	12.41	<0.001^***^
Object type * Grammatical aspect	−0.01	0.02	5517.06	−0.53	0.59
Object type * Object state	−0.06	0.02	5517.00	−2.93	<0.01^**^
Grammatical aspect * Object state	−0.02	0.02	5516.99	−0.91	0.36
Object type * Grammatical aspect * Object state	−0.01	0.04	5517.00	−0.33	0.75

*Note*. RT ∼ Object type * Grammatical aspect * Object state + (1 | participant) + (1 | item).

*** *p* < .001; ^*^* *p* < .01; ^*^ *p* < .05.

In line with the predictions of the event knowledge violation penalty, we found an interaction between object state and object type (*p* < .01; Table 2). Planned comparisons showed that this was driven by differential effects of object type in state‐change images no‐state‐change images. Within state‐change images (right side of Fig. 1), we found an effect of object type (*p* < .01): RTs to unlikely state‐change images were longer than RTs to likely state‐change images (Table 3). Within no‐state‐change images (left side of Fig. 1), there was no effect of object type (*p* = .33; Table 4). This supports our hypothesis that images depicting unlikely state changes will elicit a processing cost, that is, the event knowledge violation penalty.

**Table 3:** Planned comparisons, state‐change images only

	*β*	*SE*	df	t value	*Pr* (>|*t*|)
(Intercept)	6.48	0.05	77.63	119.51	<0.001^***^
Object type	−0.05	0.02	2620.93	−3.10	<0.01^**^
Grammatical aspect	0.03	0.02	2620.97	1.99	0.047 ^*^
Object type * Grammatical aspect	−0.02	0.03	2620.98	−0.52	0.60

*Note*. RT ∼ Object type * Grammatical aspect + (1 | participant) + (1 | item).

*** *p* < .001;^*^* *p* < .01; ^*^ *p* < .05.

**Table 4:** Planned comparisons, no‐state‐change images only

	β	SE	df	t value	Pr (>|t|)
(Intercept)	6.35	0.04	98.25	143.79	< 0.001^***^
Object type	0.01	0.01	2633.94	0.97	0.33
Grammatical aspect	0.05	0.01	2634.01	3.84	<.001^***^
Object type * Grammatical aspect	−0.01	0.03	2634.02	−0.19	0.85

*Note*. RT ∼ Object type * Grammatical aspect + (1 | participant) + (1 | item).

*** *p* < .001; ^*^* *p* < .01; ^*^ *p* < .05.

To address the question of how the grammatical aspect modulates (initial and end) object state representations, we examined whether there is an effect of the grammatical aspect in no‐state‐change images and in state‐change images. We found that RTs to no‐state‐change images (initial states) were longer when the sentence was presented in the imperfective aspect, compared to the perfective aspect (*p* < .001). This was also the case with state‐change images (end states): images were processed faster in the perfective aspect, compared to the imperfective aspect, sentences (*p* < .05). In other words, both initial and end states were processed faster in the perfective aspect, compared to the imperfective aspect, sentences.

Finally, to investigate whether the perfective aspect amplifies the event knowledge violation penalty, we looked for an interaction between the grammatical aspect and object type specifically within the state‐change image conditions. While there was an effect of object type (*p* < .01), we found no interaction (*p* = .60) between the grammatical aspect and object type within state‐change image conditions (Table 3). Thus, the event knowledge violation penalty persists in both the perfective and imperfective sentences—that is, regardless of the grammatical aspect.

## 3 General discussion

This study investigated how real‐world event knowledge contributes to object state representations when the event is described as ongoing or completed. Research on the role of real‐world event knowledge during language comprehension has often focused on which event participants are involved. However, an important facet of event knowledge is knowledge about the state‐changes that event participants undergo. We investigated whether and how event knowledge about (un)likely state‐changes influences object state representations during sentence comprehension. Our results revealed that real‐world knowledge about events influences object state representations, such that integration of unlikely state‐change information is costly (the event knowledge violation penalty). This was reflected in RT slowdowns in sentences describing potential state‐changes. In other words, even in the presence of linguistic input evoking event structures where state‐change is possible, participants’ RTs to state‐change images were modulated by object properties (i.e., how likely they are to undergo state‐change).

As we noted above, given that our real‐world event knowledge about the likelihood of state‐change is related to real‐world knowledge, including sensory experience (e.g., visual experiences; how often we see objects in changed vs. unchanged states), our results can be regarded as reflecting both abstract real‐world knowledge about state‐change likelihood, as well as the ease of recognizing visual depictions of likely versus unlikely state‐changes for different objects.

Moreover, we extend prior work showing that perfective aspect focuses attention on event boundaries, while imperfective aspect shifts attention away from it (Madden‐Lombardi et al., 2017; Magliano & Schleich, 2000; Misersky et al., 2021). Our data indicate that perfective aspect increases the accessibility of both the end state and, notably, the initial state of an event. While prior research has linked perfective aspect to end‐state activation, it has not shown similar effects for initial states. We provide new evidence that perfective aspect can highlight both boundaries of an event—its beginning and its end. This finding aligns with the view that the perfective aspect promotes a holistic representation, drawing attention to both boundaries.

Overall, our data do not provide evidence that the strength of the event knowledge violation penalty is modulated by grammatical aspect. The processing penalty associated with unlikely state changes occurs regardless of whether the event is described as completed, as signaled by perfective aspect, or ongoing, as signaled by imperfective aspect.

These results suggest that language comprehension involves predictions about described events, such as the outcomes of the ongoing action, and that these predictions are modulated by event knowledge. Additionally, by using an online self‐paced reading paradigm with images, we were able to observe such effects in real‐time during comprehension. These results suggest that object state representations are dynamically updated as the comprehender incrementally processes linguistic information about the event being described. These conclusions are compatible with the idea that predictions during language processing are mediated by event knowledge (e.g., knowledge about event participants) and that this knowledge is automatically activated in real‐time (e.g., Altmann & Kamide, 1999; Kim, Oines, & Sikos, 2016). The question of whether and how prior linguistic context about event participants can interact with general real‐world knowledge to mediate the likelihood of state‐change is an intriguing direction for future work. More generally, future work can shed further light on the scope and nature of predictions during event comprehension.

The nature and time course of the interaction between event knowledge and grammatical aspect cues may differ across languages, depending on factors such as how a language encodes aspectual information (e.g., by means of verb morphology, case marking, particles, adverbs), what kind of aspectual distinctions are encoded in a particular language, the word order patterns of the language (which can impact when aspectual information is encountered during real‐time processing), and the rigidity and reliability of these patterns. We investigated English, an Subject‐Verb‐Object (SVO) language that encodes perfective/imperfective aspect in the verbal domain, because these properties make it well‐suited for exploring how information carried by the verb influences object state representations. There are many intriguing questions regarding the interaction between event knowledge and grammatical aspect cues that merit further work, especially in verb‐initial and verb‐final languages, languages that encode aspect in different ways, as well as languages where (some) aspectual distinctions are not grammatically encoded (see also related work by Bott & Hamm, 2014; Bott & Gattnar, 2015, on German, Russian, and English). A better understanding of how these cross‐linguistic differences interface with real‐world knowledge is a worthwhile direction for future work.

As a whole, this study adds to the body of work showing that non‐linguistic information grounded in real‐world knowledge as well as linguistic cues can be used to dynamically update mental representations and potentially their visual instantiations. These findings provide novel insights into how linguistic input is mapped onto event representations during online language comprehension. Building mental representations of how the described event unfolds over time—that is, understanding the change(s) that the objects undergo—is a process that involves rapidly integrating general semantic knowledge about events and their outcomes in the world.

## Data Availability

All raw data and the code behind the analyses have been made available on the Open Science Framework (OSF) and can be accessed at: https://osf.io/rgx2a/.
